# Impact of Nutritional Supplements on the Fitness of the Parasitoid *Binodoxys communis* (Gahan)

**DOI:** 10.3390/insects15040245

**Published:** 2024-04-03

**Authors:** Wanhong Hu, Bing Liu, Shike Xia, Ningwei Ma, Peiling Wang, Yanhui Lu

**Affiliations:** 1 Xinjiang Production and Construction Corps Key Laboratory of Special Fruits and Vegetables Cultivation Physiology and Germplasm Resources Utilization/Key Laboratory of Oasis Agricultural Pest Management and Plant Protection Resources Utilization, Xinjiang Uygur Autonomous Region/College of Agriculture, Shihezi University, Shihezi 832003, China; huboss0424@163.com (W.H.); 15999299650@163.com (N.M.); 2 State Key Laboratory for Biology of Plant Diseases and Insect Pests, Institute of Plant Protection, Chinese Academy of Agricultural Sciences, Beijing 100081, China; liubing1945@126.com (B.L.); xiashikee@163.com (S.X.); 3 Western Agricultural Research Center, Chinese Academy of Agricultural Sciences, Changji 831100, China

**Keywords:** *Binodoxys communis*, fecundity, fitness, flight ability, longevity, parasitism, parasitoid behavior, sugar

## Abstract

**Simple Summary:**

Different nutritional supplements can differentially affect the reproductive performance of parasitoid wasps, and selection of optimal diets can increase parasitoid quality, increasing their effectiveness as biological control agents. *Binodoxys communis* (Gahan) (Hymenoptera: Aphelinidae) is the main parasitoid attacking *Aphis gossypii* Glover (Hemiptera: Aphidae) in Xinjiang, China. In this study we tested the effects of fructose, glucose, sucrose, trehalose, maltose, melezitose, and sorbitol on the longevity, parasitism ability, parasitism behavior, and flight ability of *B. communis*. We found that, compared with other sugars, B. communis fed on glucose, sucrose, or fructose had significantly greatly longevity and parasitism ability. Also, parasitoids that were fed these sugars interacted with *A. gossypii* more frequently during parasitization and had an enhanced ability. The above information helps in the evaluation of nutritional supplements, both to improve the parasitoid’s reproductive performance and its effectiveness as a biological control agent.

**Abstract:**

Alterative nutritional foods consumed by adult parasitoids play an important role in their fitness and ability to control pests because of food scarcity in many crops. While adult parasitoids feed on various sugars, they vary in their nutritional value for parasitoids. We assessed the effects of seven sugars (fructose, glucose, sucrose, trehalose, maltose, melezitose, and sorbitol) on the longevity, parasitism ability, parasitism behavior, and flight ability of *B. communis*, an important parasitoid of cotton aphids. We found that access to glucose, sucrose, or fructose, increased *B. communis* adult longevity more than the other sugars offered. All sugars except trehalose increased the parasitism rate to more than 50% compared to the starved control (only provided with water). We then compared parasitoid behaviors of wasps fed glucose, sucrose, or fructose to that of the starved control (with access only to water) and found that those fed *B. communis* spent more time either examining or attacking aphids than parasitoids in the control group, which spent more time walking or resting. Also, consumption of glucose, sucrose, or fructose also significantly improved the flight ability (the total flight distance, flight time, and average flight speed) of *B. communis*.

## 1. Introduction

In agricultural ecosystem, access to food supplements improves the effectiveness of parasitoid wasps, improving their performance as biological control agents. Nutritional supplements can significantly extend the longevity of parasitoid wasps, promote oogenesis, and increase parasitism, improving parasitoid reproduction [1,2,3,4]. In agricultural ecosystems, the quality and availability of resources for adult parasitoids vary greatly over time and space. Food deprivation or feeding on poor quality foods can affect the longevity, parasitism rate, and parasitic behavior of female parasitoids, reducing their potential impact as biological control agents [5,6].

Parasitoid wasps feed on nectar, pollen, or honeydew to meet their nutritional needs [7]. Plant nectars (both floral and extrafloral) are mainly composed of glucose, fructose, sucrose, and amino acids, foods that can be quickly digested by parasitoid wasps, and that are well known to extend the longevity of adult parasitoids [8]. Other studies have shown that pollen also acts as a sugar source. For example, feeding on corn pollen significantly extended the longevity of *Trichogramma brassicae* (Bezdenko) (Hymenoptera: Trichogrammatidae) [9]. Honeydew excretions of Hemiptera are also sugar-containing liquids, but honeydews of different origins can vary greatly in their sugar content [10]. However, some of the oligosaccharides in honeydew cannot be metabolized by parasitic wasps [11]. Wyckhuys et al. [12] reported that the parasitoid *Binodoxys communis* (Gahan) that fed on the honeydew of soybean aphid, *Aphis glycines* (Matsumura), had a shorter longevity compared to fed on honey or sucrose, due to the total sugar and glycogen levels of honey or sucrose-fed individuals were consistently higher than those fed honeydew or water. Indeed, some honeydews are of a poorer quality as sugar sources for parasitoids than nectars or defined sugar solutions [13,14,15]. Availability and quality of food resources can play a key role in parasitoid–host dynamics [5]. Zhang [16] studied the effects of six carbohydrates (fructose, glucose, sucrose, trehalose, raffinose, and honey) on the longevity, egg-laying performance, and nutritional level of the braconid *Meteorus pulchricornis* (Wesmael), when honey or sucrose solutions were provided, female parasitoid wasps produced more offspring and had greater longevity than parasitoids fed other sugar diets or controls. Similarly, the ichneumonid *Diadegma semiclausum* (Hellén) of *Plutella xylostella* (L.) preferred various sugar sources such as trehalose, sucrose, melibiose, fructose, maltose, glucose, and honeydew, which can significantly prolong the life span, whereas raffinose, lactose, and melezitose as well as the water control group had no positive effect on the adult longevity [17]. Zamek et al. [18] found that white sugar gave the highest adult female lifespan of a fruit fly (*Bactrocera tryoni* Froggatt) parasitoid, *Diachasmimorpha tryoni* (Cameron), while honey (10% concentrations) and golden syrup (56% invert syrup (glucose and fructose), 44% sucrose) shared similar survivorship curves; they were all significantly greater compared with water control females.

In addition to increasing longevity and fecundity, sugar feeding can also affect other biological functions of parasitoids, such as foraging, host detection, and flight ability. For example, the searching efficiency of primary parasitoid *Aphidius ervi* (Haliday) of pea-aphid (*Acyrthosiphon pisum* Harris) was enhanced by access to floral nectar of buckwheat [19]. Similarly, buckwheat nectar-fed female parasitoid *Diaeretiella rapae* (M’Intosh) showed enhanced host searching ability on the aphid host *Lipaphis erysimi* (Kaltenbach) when compared to the water-fed control [2]. When fed on the floral nectar of *Anethum gravolens* (L.), the female wasps *Cotesia glomerata* (L.) (Hymenoptera: Braconidae), which is a gregarious larval parasitoid of *Pieris brassicae* (L.) performed flight activity, as well as those feeding on honey, which was superior to those feeding on sucrose; feeding on the floral nectar of *Origanum vulgare* (L.) has a similar effect to feeding on the extrafloral nectar of *Vicia faba* (L.) and significantly increased the total distance flown but not the number of flights or the longest single flight [20]. Providing a high-quality diet suitable for the parasitoids can maximize the reproductive capacity of parasitoids throughout their lives, and improve the effectiveness of biological control [21,22]. For instance, when *Aphytis melinus* (DeBach) were released with artificial sugars spray in the field, the fecundity and parasitism rate on the *Aonidiella aurantii* (Maskell) host was higher than the water control and the release without sugars [23]; Xia et al. [24] reported fructose and glucose in buckwheat nectar could significantly enhance *Peristenus spretus* (Chen and van Achterberg) survival and parasitism of the mirid *Apolygus lucorum* (Meyer-Dür) nymphs compared to the water control.

In recent years, *Aphis gossypii* (Glover) has become the main pest affecting cotton yields in Xinjiang province China [25]. *B. communis* is the dominant parasitoid of cotton aphids, accounting for >95% of the primary parasitoid communities in this region [26]. In a diversified agricultural landscape, natural enemies such as parasitoids can benefit from crop or non-crop habitats which supply as flowering or nectariferous plants, thus improve their fitness and parasitism on crop pests [27,28,29,30]; however, agricultural intensification usually reduces the proportion of (semi-)natural habitats which play a critical role in conservation biological control [31]. In order to buffer these negative ecological effects, artificial-provided nutrition such as sugar sprays or cultivate flowering bank vegetations could be an effective solution, especially when the diversity or ecosystem service functions are in short supply [23,32,33]. Although the local agricultural ecosystem in Xinjiang is highly intensive, it may potentially fail to provide sufficient resources or refuges for natural enemies, such as parasitoid wasps due to a low-level habitat diversity in this cotton production region. To restore needed resources in this cropping region, information on the value of particular sugars or other nutrition (and their availability in functional companion plant nectars) on the key aphid parasitoids is essential. In this study, we aimed to assess the effects of different carbohydrate food sources (various sugars) on the longevity, parasitism, parasitoid behavior, the flight ability of *B. communis*, and to develop a suitable tactic for the conservation of this parasitoid enemies, thus improving their biological pest control services for cotton aphids.

## 2. Materials and Methods

### 2.1. Insect Rearing

Cotton aphids *A. gossypii* and their mummies were collected from cotton fields at the Korla Experimental Station (41.75° N, 85.81° E), Chinese Academy of Agricultural Sciences (Korla city in Xinjiang province). *A. gossypii* was reared on young cotton leaves and maintained in screened cages (60 × 40 × 50 cm). *B. communis* adults that emerged from the mummies were identified and reared by placing adults in one of the *A. gossypii* rearing cages stocked with aphids. Both of these insect colonies were held at 26 ± 1 °C, 70 ± 5% RH and 16:8 h (L:D) photoperiod. After the *B. communis* population had stabilized (about four weeks), aphid mummies were collected from the cotton leaves when needed for experiments. Mummies were held in a plexiglass box (30 × 30 × 30 cm) for adult emergence, and the newly emerged adults were used for experiments.

### 2.2. Effect of Nutritional Supplements on Longevity of B. communis

We tested the nutritional values of seven compounds (all ≥ 98% pure; all purchased from Coolaber Science and Technology Co., Ltd., Beijing, China, except D(+)-Melezitose monohydrate, which was purchased from InnoChem, Beijing, China): (1) D-glucose, (2) D-fructose, (3) sucrose, (4) D(+)-trehalose dihydrate, (5) D(+)-maltose monohydrate, (6) D(+)-melezitose monohydrate, and (7) D-sorbitol, with water as the control. Newly emerged *B. communis* were reared individually in small round plastic chambers (mouth diameter 6.5 cm, bottom diameter 5.5 cm, height 3.4 cm). Simultaneously, for each treatment group, cotton balls were soaked with 3 mL of a 1 M solution of each test compound and then placed in the test containers as food for *B. communis* adults. The test chambers were ventilated by several holes (1 mm dia) that were screened to prevent parasitoid escape. Every treatment included at least 40 wasps of each sex. Every 12 h, the numbers of dead males and females were recorded, all dead individuals removed, and cotton balls replaced. This process was repeated until all *B. communis* in each treatment had died. All tests were run in a controlled climate chamber (RXZ500D, Jiangnan Instrument Factory, Ningbo, China) at 25 ± 1 °C, 60 ± 10% RH, and a 16:8 (L:D) photoperiod.

### 2.3. Effects of Nutritional Supplements on Parasitism by B. communis

To determine the effect of the nutritional supplements on parasitism by *B. communis*, we first placed 30 adult cotton aphids in a 12-well plate with cotton leaves in the wells, which carpeted with 10% water–agar jelly for leaf moisturizing. After these adults had produced nymphs within 24 h, 60 s instar nymphs of similar size, 5 per well plate, were retained, and all other aphids and excess nymphs were removed. Well plates with 2nd instar nymphs were then placed in square plastic insect-rearing containers, together with a pair of 2-day-old adults of *B. communis*. The top of the square plastic container was closed with gauze, and a cotton ball soaked in a water solution of one of the test nutrients was added to provide nutrition for the adult parasitoids. After 24 h, we removed the *B. communis* adults and then continued to rear the aphid nymphs exposed to parasitism. During nymphal rearing, the water–agar jelly in the wells and cotton leaves were replaced every 3 days. When replacing leaves, all the nymphs in a well were transferred by a small paintbrush onto the new cotton leaves. As this occurred, all aphid mummies were counted as the measure of the number of aphids that were parasitized in each treatment. We also counted the number of mummies from which adult parasitoids emerged; we recorded the time when aphid mummies first appeared (after the mummies were completely hardened); and we recorded the proportion of females among the F1 parasitoids that emerged from mummies (F/(M + F)). All tests were run in a controlled climate chamber (RXZ500D, Jiangnan Instrument Factory, Ningbo, China) at 25 ± 1 °C, 60 ± 10% RH, and a 16:8 (L:D) photoperiod.

### 2.4. Effects of Nutritional Supplements on Parasitoid Behaviors

We screened glucose, fructose, and sucrose, the three sugars that have the greatest impact on the longevity and parasitism of *B. communis*, and a water control to evaluate the impact of these nutritional supplements on host searching behaviors of *B. communis*. Pairs of newly emerged *B. communis* that had been fed the treatment sugars (1 M) or water only for 24 h and allowed to mate. Females were then placed individually in Petri dishes (diameter: 5.4 cm; height: 1.4 cm), together with 60 s instar cotton aphids and 3 drops of 5 μL of a particular nutrient treatment. Female parasitoids were released in the center of the Petri dish and then observed for 30 min under a Leica dissecting microscope (model S9i) to observe and record the parasitoid’s behavior. There were 5 replicates for each treatment. Tests were run in the morning (08:00 to 12:00 a.m.), which corresponded to the peak period of parasitoid activity [34]. Parasitoid behavior was recorded as discrete states: walking (host searching, walking, or exploring arena), drumming (part of host assessment), oviposition (inserting ovipositor into aphid host), grooming (cleaning body parts using legs or mouth), resting (remaining motionless), or feeding (drinking from the diet droplets). Behavioral observations were analyzed using behavioral observation research interactive software [35] to determine the duration spent in each behavioral state.

### 2.5. Effects of Nutritional Supplements on Flight Ability

To measure the effects of glucose, sucrose, fructose, and a water control (1 M) on the flight ability of male and female *B. communis*, we tested these parasitoids in an insect flight mill system. When parasitoids were newly emerged, they were fed each of the various treatment diets as described above for the flight test. In total, 25 *B. communis* individuals of each sex were tested for each treatment. Wasps were tested using the Jiaduo Internet of Things Insect Flying Mill Information System (Hebi Jiaduo Weinong Agriculture and Forestry Technology Co., Ltd., product model: FXM-Z, Hebi City, China).

The flight mill consisted of a computer, a sensor, a communication line, a flight arm (composed of copper wire that was 10 cm long and 0.01 cm in diameter), a No. 3 insect pin (clipped off at the upper part of the pin), light-shielding paper, and a gold wire (15 μm in diameter) used for insect bonding. The flying device used the insect pin as the flight axis. The upper part of the flight axis had a flight arm with a radius of 5 cm. The end of the flight arm had a 2 cm gold wire attached in parallel to the flight axis. The lower end of the flight axis was a piece of light-shielding paper (1.5 cm long by 0.5 cm wide). To set up the test, an ice bag was placed under an insect dissecting microscope, and a parasitoid was placed on the ice bag to immobilize it. We then placed a small amount of glue (product number 5201, Shenzhen Kingsbond Adhesive Co., Ltd., Shenzhen City, China) at the end of a gold wire used to bond the insect to the mill. Once the wire was firmly glued to the prothorax of the parasitoid, the wasp was allowed to rest for 4 to 5 s while the glue set. After making sure that the glue had not damaged the parasitoid’s wings or flight muscles, we placed the flight axis vertically into a flight mill with magnets above and below. Since only the tip of the needle is in contact with the magnet, any friction was minimal. We then allowed the wasp to fly in a circle around the flight axis. The flight system recorded the number of flight circles by sensing the parasitoid that was driving the rotation of light-shielding paper at the lower end of the flight axis. The distance of one flight circle was 31.42 cm. Any *B. communis* wasps that showed an abnormal wingspan or flight behavior were excluded from the test.

Flight was continuously monitored for 24 h for each wasp, recording the time each flight was initiated and ended, and the number of times of flight circles completed in consecutive 10 s intervals. The number of flight mill revolutions was used to compute flight distance, speed, and duration for each wasp. Flight tests were run at 25 ± 2 °C, a 12:12 (L:D) photoperiod, light intensity of 1.22 klux, and no wind disturbance.

### 2.6. Data Analysis

Analysis of the survival rates of *B. communis* fed on different diets was conducted using Cox regression using the “survifit” function within the “survival” package [36]. One-way analysis of variance (ANOVA) was used to compare the longevity, parasitism rate, mummy emergence rate, number of days before complete mummification of first aphid, the proportion of offspring parasitoids that were females (F/(F + M)), the duration of each parasitic behavior during the parasitization process, flight time, flight speed, and flight distance between different treatments. We then used Tukey’s HSD test at the 0.05 level for multiple mean comparisons. Before data analysis, the original data were log_10_-transformed to ensure normality and homogeneity of variances. All analyses were carried out with R 4.3.1 software [37]. Figures were prepared using Graphpad Prism 9.5 software.

## 3. Results

### 3.1. Effects of Nutritional Supplements on the Longevity of B. communis

**Figure 1:**
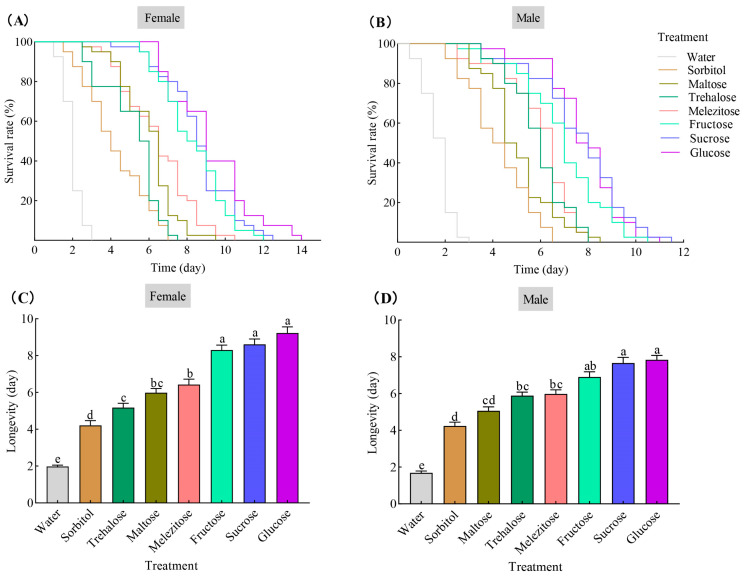
Survival curves of female (**A**) and male (**B**) *B. communis* fed with different sugar sources and the mean (±SE) survival time of females (**C**) and males (**D**). Means followed by the same lowercase letters are not statistically different (*p* > 0.05, Tukey HSD). The sample size of female and male *B. communis* was 40 in each treatment, respectively.

Cox regression analysis showed that feeding on different diets significantly affected the survival rate of female (χ^2^ = 362.43, df = 7, *p* < 0.001) (Figure 1A) and male (χ^2^ = 342.51, df = 7, *p* < 0.001) (Figure 1B) *B. communis*, compared to the water control; being fed on sugars significantly prolonged the survival duration of this parasitoid, regardless of being female or male adults.

The longevity of female *B. communis* under nutritional supplementation was significantly varied (*F*_7,312_ = 120.98, *p* < 0.001), and the longevity of all sugar treatments were longer than those fed on water control (1.98 ± 0.08 d). Females fed on glucose had the longest longevity (9.23 ± 0.33 d), then followed by those fed on sucrose (8.61 ± 0.29 d) and fructose (8.30 ± 0.27 d); these were longer than those fed on melezitose (6.43 ± 0.30 d), maltose (5.99 ± 0.23 d), trehalose (5.18 ± 0.23 d), and sorbitol (4.21 ± 0.26 d), respectively (Figure 1C).

Similar to the female parasitoids, the longevity of male *B. communis* under different nutritional supplementation was also significantly varied (*F*_7,312_ = 111.35, *p* < 0.001), and the longevity by all of the sugar treatments was longer than those fed on water control (1.69 ± 0.10 d). Male adults fed on glucose (7.84 ± 0.24 d), sucrose (7.66 ± 0.31 d), and fructose (6.90 ± 0.29 d) had higher longevity, the following highest were fed on melezitose (5.98 ± 0.23 d), trehalose (5.89 ± 0.19 d), and maltose (5.06 ± 0.22 d), all of these significantly prolonged the male longevity than feeding on sorbitol (4.24 ± 0.21 d) and water control (Figure 1D).

### 3.2. Effects of Nutritional Supplements on Parasitism of B. communis

The days until the mummies appeared showed no significant difference between different treatments (all of them need about 4–5 days, *F*_7,32_ = 0.52, *p* = 0.816). However, the parasitism rate was significantly affected by different treatments (*F*_7,32_ = 32.96, *p* < 0.001). *B. communis* fed on glucose had the highest parasitism rate at 69.54 ± 3.34%, when provided with melezitose, maltose, sucrose, and fructose, the parasitism rates were also higher, and they were significantly higher than trehalose treatment (43.39 ± 0.76%), and the water control (26.8 ± 1.27%). The emergence rates of parasitoid offspring from mummies were higher under sucrose (81.13 ± 2.47%), melezitose (74.97 ± 2.39%), and fructose (73.55 ± 1.78%) treatments, compared to only 41.06 ± 3.48% in the water control (*F*_7,32_ = 35.36, *p* < 0.001). The proportion rates of female offspring ranged from 49.9–62%, which had no significant difference between different treatments (*F*_7,32_ = 0.44, *p* = 0.868) (Table 1).

### 3.3. Effects of Nutritional Supplements on Parasitoid Behaviors

**Figure 2:**
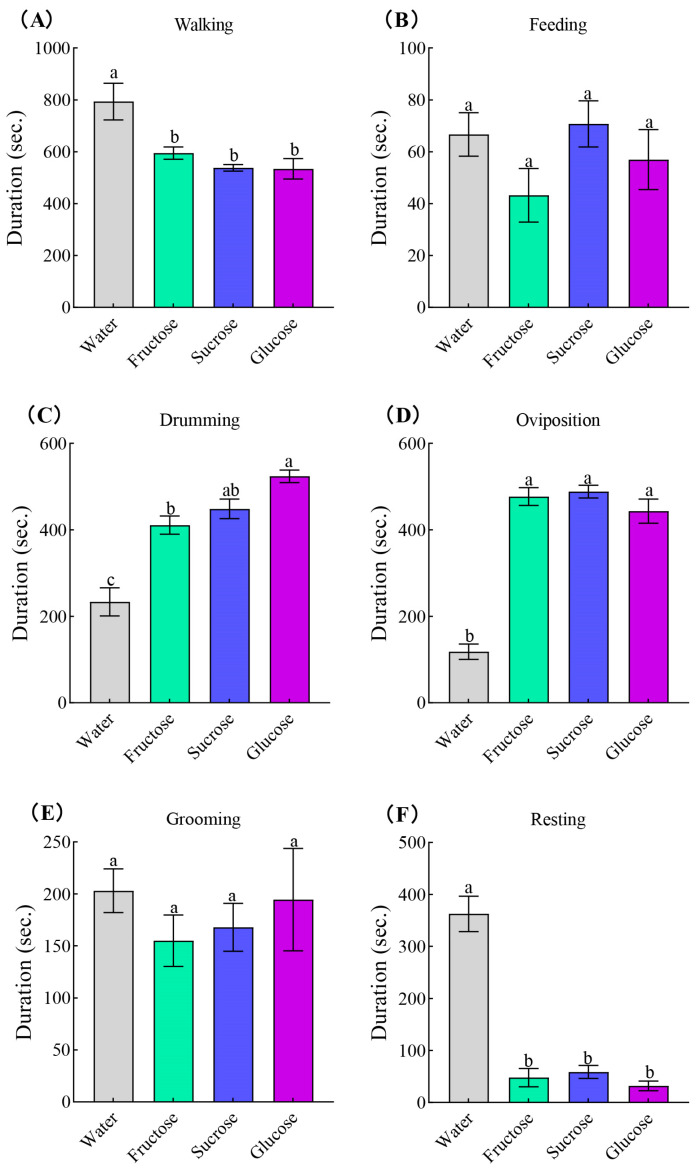
Duration of behavioral states of *Binodoxys communis* adults fed different diets. Observations were made on parasitoids that had after parasitized an *Aphis gossypii* nymph within the previous 30 min (mean ± SE). Means followed by the same lowercase letters are not statistically different (*p* > 0.05, Tukey HSD). (**A**), walking; (**B**), feeding; (**C**), drumming; (**D**), oviposition; (**E**), grooming; (**F**), resting.

Sugar feeding also affected parasitoid behaviors. During the 30 min observation period, females fed on glucose, fructose, or sucrose spent less time walking (*F*_3,16_ = 7.75, *p* = 0.002, Figure 2A) and resting (*F*_3,16_ = 18.02, *p* < 0.001, Figure 2F) than water control treatment. The durations of drumming (*F*_3,16_ = 18.66, *p* < 0.001, Figure 2C) and oviposition (*F*_3,16_ = 59.82, *p* < 0.001, Figure 2D) under sugar treatments were significant longer than the water control. However, the time that females spent on grooming (*F*_3,16_ = 0.61, *p* = 0.617, Figure 2E) and feeding (*F*_3,16_ = 1.76, *p* = 0.196, Figure 2B) had no significant difference within different treatments.

### 3.4. Effects of Nutritional Supplements on Flight Ability

Nutritional supplements significantly improved the flight ability of *B. communis*, such as the flight speed (female: *F*_3,96_ = 5.17, *p* = 0.002; male: *F*_3,96_ = 8.90, *p* < 0.001), the total flight time (female: *F*_3,96_ = 46.98, *p* < 0.001; male: *F*_3,96_ = 59.22, *p* < 0.001), and flight distance (female: *F*_3,96_ = 119.79, *p* < 0.001; male: *F*_3,96_ = 107.55, *p* < 0.001), compared to water control. For female and male adults fed on sucrose had the highest average flight speed, whereas feeding on glucose had the longest flight time and flight distance (Table 2).

## 4. Discussion

The effectiveness of parasitoids as biological control agents can be improved by sugar feeding, which extends longevity, adult dispersal, and reproductive capacity [32,38,39]. During periods of food scarcity, parasitoid wasps may spend more time searching for food and reabsorb their eggs to extend their survival. We examined the effects of different sugars on the longevity, the level of parasitism, key parasitism behaviors, and flight ability of *B. communis*. We found that food supplementation with different sugar sources can prolong the longevity, reproduction, and dispersal ability of parasitoids, as observed in earlier studies.

The range of glycogen substances available to insects varies widely [5]; added sugar resources extended the longevity of parasitoids [16,40]. However, *B. communis* showed differences in longevity when fed with different sugars; they lived significantly longer when fed honey or sucrose than honeydew of soybean aphid host *A. glycines*, while starved wasps had the shortest lifespan [12]. In our study, providing sugar sources effectively improved the survival rate and longevity of aphid parasitoids. Similar results have been reported for several other parasitoid wasps, such as *Cotesia vestalis* (Haliday) (Hymenoptera: Braconidae) [41] and *Psyttalia concolor* (Szepligeti) (Hymenoptera: Braconidae) [3]. We found when *B. communis* was fed fructose, glucose, sucrose, trehalose, maltose, melezitose, or sorbitol, their average longevity was 4–9 days, and the maximum longevity was 11 days for males and 14 days for females, whereas regarding wasps fed only water survived only 1–3 days, the longevity was greater on glucose, sucrose, and fructose than on other sugar sources.

In our study, wasps fed with sugars and offered hosts attacked 54–69% of hosts offered, except for trehalose. Wasps fed only water in our study attacked only 26% of the hosts offered. Trehalose is a rapidly crystallizing sugar, which might make it more difficult for *B. communis* to absorb the sugar. Overall, providing parasitoid females with an optimal sugar-rich diet clearly increases egg-laying behavior [42].

Behavioral tests with parasitoids fed on glucose, sucrose, or fructose showed that sugar-fed parasitoids spent more time in drumming and oviposition behaviors, while starved parasitoids provided with only water spent more time walking and resting, which is similar with Straser et al.’s findings for another egg parasitoid, *Hadronotus pennsylvanicus* (Ashmead) [6]. Some studies, however, have shown that starvation can enhance parasitoid egg-laying behavior, causing wasps to lay their eggs more rapidly, improving their fitness. For example, Takano et al. [43] found that starved females of the egg parasitoid *Paratelenomus saccharalis* (Dodd) (Hymenoptera: Platygastridae) laid their eggs faster and more often than well-fed females. Their study suggests that under some conditions, when adult mortality is likely to be high, parasitoids may show a preference to reproduce as quickly as possible. This variation in the effect of sugar supplements on parasitoids may be due to differences in the parasitoid species tested [1,44,45].

Feeding on glucose, sucrose, or fructose increased the flight ability of *B. communis*. Flight times and distances of female *B. communis* feed glucose, sucrose, or fructose were significantly greater than individuals given only water. However, among the sugars tested, *B. communis* showed little difference in flight speed. Parasitoids in our test used supplemental sugars, in particular glucose, sucrose, or fructose to maximize their flight activities.

Under field conditions, the availability of carbohydrate-rich resources can vary widely. Parasitoid wasps may experience periods with no access to an appropriate diet, which may reduce their fitness and the efficacy of biocontrol programs. Provision of supplemental sugars for parasitoids may enhance biological control in an integrated pest management system [32,39]. For instance, artificial sugar sprays in the field improved the fecundity and parasitism rate of *A. melinus* on the *A. aurantii* host [23]. Our study showed that glucose, fructose, and sucrose best improved the longevity, reproductive capacity, and flight ability of *B. communis*, and may be useful as food sources for this parasitoid for control of *A. gossypii* in cotton fields. Based on this, we can maximize the adaptability of parasitoid wasps by artificially spraying sucrose, glucose, and fructose solutions when releasing adult parasitoids during pest outbreaks. Additionally, we can design various bank crop or habitat configurations, such as flowers, pollen, nectar, and insect honeydew, to maintain the high availability and utilization of carbohydrate resources by parasitoids. These strategies will promote parasitoid conservation and enhance biocontrol services.

## Figures and Tables

**Table 1:** The impact of nutritional supplements on parasitism of *Aphis gossypii* by *Binodoxys communis*.

Treatment	Days Until Mummy Appeared (d)	% Parasitism	% Mummy Emergence	% Female Progeny
fructose	4.20 ± 0.12 a	58.51 ± 1.16 ab	73.55 ± 1.78 ab	61.94 ± 10.45 a
glucose	4.10 ± 0.19 a	69.54 ± 3.34 a	63.73 ± 2.79 bc	62.37 ± 6.29 a
sucrose	4.10 ± 0.19 a	62.85 ± 3.13 ab	81.13 ± 2.47 a	51.9 ± 2.63 a
trehalose	4.10 ± 0.19 a	43.39 ± 0.76 c	61.09 ± 1.16 dc	52.02 ± 7.39 a
maltose	4.10 ± 0.19 a	65.35 ± 1.97 ab	50.96 ± 2.07 de	59.68 ± 6.35 a
melezitose	4.10 ± 0.19 a	66.45 ± 2.61 a	74.97 ± 2.39 a	49.9 ± 10.78 a
sorbitol	4.30 ± 0.30 a	54.58 ± 3.87 bc	52.29 ± 1.54 d	60.44 ± 4.44 a
water	4.50 ± 0.22 a	26.8 ± 1.27 d	41.06 ± 3.48 e	58.38 ± 8.18 a

Data are mean ± SE; means followed by the same lowercase letters are not statistically different (*p* > 0.05, Tukey HSD).

**Table 2:** Flight ability of *Binodoxys communis* fed different nutritional supplements.

Treatment	Flight Speed (m/h)		Flight Time (/h)		Flight Distance (/m)	
	Female	Male	Female	Male	Female	Male
glucose	284.10 ± 6.41 a	287.19 ± 6.93 a	2.72 ±0.06 a	2.48 ± 0.06 a	767.10 ± 13.54 a	708.65 ± 18.43 a
sucrose	290.46 ± 6.43 a	307.22 ± 6.16 a	2.64 ± 0.08 a	2.12 ± 0.07 b	759.37 ± 19.52 a	647.01 ± 19.79 a
fructose	271.59 ± 8.11 ab	285.47 ± 6.75 a	2.41 ± 0.14 a	1.89 ± 0.10 b	638.84 ± 30.91 b	535.08 ± 27.07 b
water	249.87 ± 11.73 b	252.72 ± 10.19 b	1.43 ± 0.10 b	1.14 ± 0.05 c	338.19 ± 13.54 c	279.56 ± 9.70 c

Data are means ± SE; means followed by the same lowercase letters are not statistically different (*p* > 0.05, Tukey HSD).

## Data Availability

All data analyzed in this study are included in this article.
